# Role of environmental specificity in CASP results

**DOI:** 10.1186/s12859-023-05559-8

**Published:** 2023-11-11

**Authors:** Irena Roterman, Katarzyna Stapor, Leszek Konieczny

**Affiliations:** 1 Department of Bioinformatics and Telemedicine, Jagiellonian University - Medical College, Medyczna 7, 30-688 Krakow, Poland; 2 Faculty of Automatic, Electronics and Computer Science, Department of Applied, Informatics, Silesian University of Technology, Akademicka 16, 44-100 Gliwice, Poland; 3 Jagiellonian University - Medical College, Kopernika 7, 31-034 Krakow, Poland

**Keywords:** Protein folding, Folding simulation in Silico, Folding environment, CASP

## Abstract

**Background:**

Recently, significant progress has been made in the field of protein structure prediction by the application of artificial intelligence techniques, as shown by the results of the CASP13 and CASP14 (Critical Assessment of Structure Prediction) competition. However, the question of the mechanism behind the protein folding process itself remains unanswered. Correctly predicting the structure also does not solve the problem of, for example, amyloid proteins, where a polypeptide chain with an unaltered sequence adopts a different 3D structure.

**Results:**

This work was an attempt at explaining the structural variation by considering the contribution of the environment to protein structuring. The application of the fuzzy oil drop (FOD) model to assess the validity of the selected models provided in the CASP13, CASP14 and CASP15 projects reveals the need for an environmental factor to determine the 3D structure of proteins. Consideration of the external force field in the form of polar water (Fuzzy Oil Drop) and a version modified by the presence of the hydrophobic compounds, FOD-M (FOD-Modified) reveals that the protein folding process is environmentally dependent. An analysis of selected models from the CASP competitions indicates the need for structure prediction as dependent on the consideration of the protein folding environment.

**Conclusions:**

The conditions governed by the environment direct the protein folding process occurring in a certain environment. Therefore, the variation of the external force field should be taken into account in the models used in protein structure prediction.

## Introduction

The protein folding problem has a long history of identification of the phenomenon in which proteins adopt a 3D structure in an explicitly defined and reproducible way [1, 2]. Monitoring the progress in this area is made possible by the CASP (“Critical Assessment Structure Prediction”) project, which has been held biannually since 1994 [3–12]. The project organiser provides participants with the amino acid sequence of a protein with a 3D structure (the target) known only to the organisers. Based on the protein sequence provided, CASP participants predict the 3D structure of the protein (model) using methods they developed [3]. In addition to the traditionally used homology-based technique, ab initio techniques are also being developed. The homology-based technique consists in finding proteins with a sequence that has a sufficiently high degree of similarity and under the assumption that a similar sequence provides a similar structure (especially if homologous proteins are involved); it has traditionally provided better results in assessing the degree of the structural accuracy of the models [13–15]. The ab initio techniques seek a theoretical model without reference to known structures, and they develop tools – as one would expect – that reproduce the mechanism of the protein folding process. This is pursued by proposing different forms of force field notation, the presence of which for a given sequence directs the structuring towards the native structure [16–19]. These two techniques dominated the history of the CASP project until 2020, when the spectacular success of artificial intelligence (AI)-based technology was reported [20]. This technique provided models of protein structures (targets) and was ranked the best in CASP in all cases. This represents a significant advance from the previous editions of CASP [21, 22].

The AI method used provides a correct structure with a high accuracy score on the GDT_TS (“Global Distance Test – Template Score”) scale used in CASP (it is the indicator used as a criterion for the degree of accuracy of model structure prediction against the target structure), which considers multiple similarity assessment criteria [3]. The AI technique uses a baseline in the form of maps of preferred inter-amino acid distances (contacts). Based on these, distances are reconstructed that are relevant to the given amino acid sequence.

However, the question of ‘Why proteins fold the way they do?’ remains unanswered. In this work, the degree of accuracy of the predicted structure was interpreted based on the consideration of the protein folding environment. The final structure of a given protein varies with the environment. Hence, using a steady-state internal force field (including preferred inter-amino acid distances) for all proteins may result in the success for one protein and a failure for another protein. The question arises as to why a jointly applied computational methods have not made any significant progress (WeFold project) [23, 24].

The Bhageerath-H server representing the hybrid ab initio/homology modelling reaching models of high accordance in CASP10 for water soluble monomeric proteins is freely available [25].

The three-track neural network linking a one-dimensional sequence level with the distance map, and the coordinate level expressing the integration of these three approaches was applied in CASP14 with good results [26].

Very popular recently techniques based on deep learning applied to the combination of template and multiple sequence alignment deliver some progress due to permanently significantly growing number of available structures making the comparable prediction more effective [27]. The interpretation of the physic-chemical 3D organisation of proteins in relation to structural and chemical classification of individual amino acids adopted to well defined homology (evolutionary) relationships is able to deliver the structures applicable for drug design techniques [28]. In the search for natural proteins sequence/structure relationships the nonnatural sequences are exploited allowing the distinguishing natural proteins on the basis of the resultant 3D structures [29]. The combination of backbone dihedral angles and relative surface accessibility for tripeptides in relation to their N- and C-terminal relative positions in 3D structures applied to machine learning techniques tested on very large diverse proteins collection seems to be promising in the development of computational methods [30].

This approach is applied in the ProFitFun-Meta server, which is freely available [31, 32].

The analysis of the reasons for the variation in the degree of accuracy of the predicted structures was based on this work on the assumption of environmental contribution in the protein structuring process. A diverse environment governs the protein folding process. The absence of a diverse environment in the protein folding model eliminates a broad spectrum of structural forms. The possibility of considering conditions imposed by the environment is offered by the fuzzy oil drop (FOD) model, as well as its modified version (FOD-M) [33, 34]. The model quantitatively assesses the contribution of the polar water environment, as well as the contribution of the diverse environment affecting the protein structure formation. This differentiation in the environment, which affects the protein folding process in a different way, is assumed to answer the question as to why the results obtained are not uniformly correct, despite the use of programs (models) that perform positively in many cases.

The ab initio technique poses the challenge of finding the mechanism of the protein folding process that leads to the prediction of the correct structure by understanding such mechanism. This work demonstrates that this is not possible if the notation of the external force field of the environment origin is not considered. Environmental differentiation – as shown by previous analyses – has a significant impact on protein structuring [35, 36]. An averaged force field (averaged parameterisation) or any other criterion notation for protein structuring in an averaged form that does not take into account the effect of external factors cannot predict the diverse structural forms of proteins. The disadvantages resulting from the averaging of the parameterisation used can be demonstrated from the results of CASP competitions. The vast majority of the tools used in the CASP project provide results of similar status by eliminating certain specific groups of proteins, where, as it turns out, the introduction of a factor in the form of a differentiated external force field is needed.

## Materials and methods

### Data

The selection of proteins analysed is limited by the condition of the availability of the protein structures acting as targets in the CASP project and the models proposed by the CASP project participants. The subjective choice was also driven by the extremes: the best and the worst results.

A comparative analysis was carried out for the following:

1. The target structures (CASP13): T0953s2-D3 (PDB ID-6F45 [37]), T0990-D3 (PDB ID-6N9Y [38]), T1024 (PDB ID – 6T1Z [39]) and models for these targets [3]. The choice of these examples was driven by the extreme (high, medium and low) accordance of models in respect to target structures using the parameters based on the FOD model as the criteria to express the effect of the environment.
2. Proteins with an all helical structure are considered to be easy. However several other factors contribute to the computational structural model ability expressed by structural difficulty (SD) taking into account secondary structures, homology and physicochemical features of protein [40]. Also the availability of suitable template structure(s) influence the quality of prediction especially in comparative modelling.
3. Proteins with the code PDB ID—6POO [41] (GDT_TS = 65.90) and the PDB target ID—6UF2 [42] (GDT_TS = 45.8), whose structure was predicted with a low level of accuracy during CASP15 [3].
4. A comparative analysis of all models provided by the same technique (Baker-Rosettaserver – participant No. 368 [43]) demonstrating its limited potential for highly accurate prediction levels. The reason for the individual failures was identified as a lack of dependence on the environment affecting the protein folding process.

The database from which the results for this analysis were taken is available at https://predictioncenter.org/ (accessed: April 26, 2023).

### Description of the FOD model

The external force field generated by the aqueous environment becomes apparent in the micellization process of bi-polar molecules, which, avoiding the entropically unfavourable contact of hydrophobic parts with polar water, form structures with an exposed polar surface, isolating hydrophobic fragments concentrated in the central part of the micelle. The description of such a hydrophobicity distribution is expressed by a 3D Gaussian function that spans the protein body:

1 $$H_{i}^{T} = \frac{1}{{H_{sum}^{T} }}\exp \left( {\frac{{ - \left( {x_{i} - \overline{x}} \right)^{2} }}{{2\sigma_{x}^{2} }}} \right)\exp \left( {\frac{{ - \left( {y_{i} - \overline{y}} \right)^{2} }}{{2\sigma_{y}^{2} }}} \right)\exp \left( {\frac{{ - \left( {z_{i} - \overline{z}} \right)^{2} }}{{2\sigma_{z}^{2} }}} \right)$$

By varying the magnitudes of the parameters σ_X_, σ_Y_ and σ_Z_, it is possible to describe globular forms of arbitrary size and shape. ‘H_i_’ expresses the idealised hydrophobicity value assuming a micelle-like system. This value assigned to the position of the effective atom (the averaged position of the atoms that make up a given amino acid) is referred to as ‘T_i_’ (theoretical).

The actual hydrophobicity distribution resulting from the inter-amino acid interaction (which depends on the distance of the interacting residues and on their intrinsic hydrophobicity) is, to a varying degree, aligned with the idealised distribution expressed by the 3D Gaussian function [33]. The determination of the actual level of hydrophobicity constituting the interaction effect is expressed by an equation proposed in [44].

2 $$H_{i}^{O} = \frac{1}{{H_{sum}^{O} }}\sum\limits_{j} {\left\{ {\begin{array}{*{20}c} {\left( {H_{i}^{r} + H_{j}^{r} } \right)\left( {1 - \frac{1}{2}\left( {7\left( {\frac{{r_{ij} }}{c}} \right)^{2} - 9\left( {\frac{{r_{ij} }}{c}} \right)^{4} + 5\left( {\frac{{r_{ij} }}{c}} \right)^{6} - \left( {\frac{{r_{ij} }}{c}} \right)^{8} } \right)} \right)} & {for\;r_{ij} \le c} \\ {0,} & {for\;r_{ij} > c} \\ \end{array} } \right.}$$

where r_ij_ is the distance between the positions of the interacting amino acids, c is the cutoff distance, and *H*^*r*^ is the intrinsic hydrophobicity. The value of the observed level of hydrophobicity (assigned to the position of the effective atom) is referred to as ‘O_i_’.

**Fig. 1:**
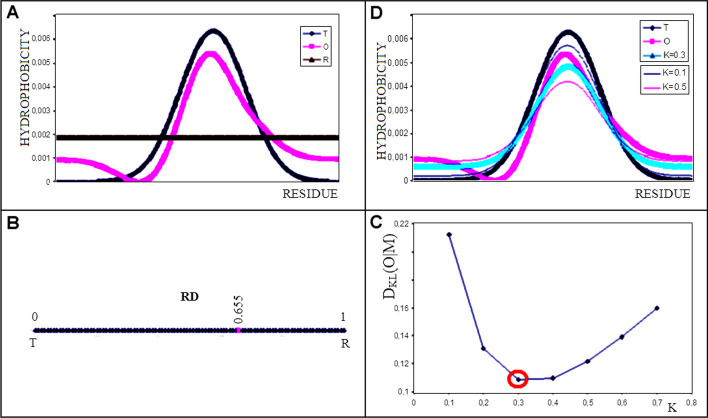
Graphical visualisation of the FOD-M model assumptions. **A** An example set of T (blue), O (pink) and R (brown) distributions. **B** The determined RD value for the example in (A) is 0.633, as shown on the axis. This value is calculated to express the difference expressed by D_KL_(O|T) and D_KL_(O|R) according to Eq. 4. **C** Determination of optimum value for K – the minimum D_KL_ value for different K values results in best fit. The K = 0.3 appears to be the optimal one for presented example. **D** The juxtaposition of the T (blue), O (pink) and M (cyan) distributions for K = 0.3 illustrates the interpretation of the M distribution, which most closely replicates the O distribution in the example in question. Additional (thin lines) represent the M distributions for K = 0.1 (thin line – pink) and M distribution for K = 0.5 (thin line – blue). The comparison of M distributions visualises the best fit for M distribution for K = 0.3

The first factor in both expressions introduces the normalisation of the distributions. The T, O and R profiles are shown in Fig. 1A.

Quantitatively, the differences between the idealised distribution, T and the actual distribution, O can be assessed using divergence entropy [45]

3 $$D_{KL} (P|Q) = \sum\limits_{i = 1}^{N} {P_{i} } \log_{2} \frac{{P_{i} }}{{Q_{i} }}$$

where P denotes the distribution under analysis (here, O) and Q denotes the reference distribution (here, T).

The D_KL_ value cannot be interpreted quantitatively. Therefore, a second reference distribution, R was introduced, where Ri = 1/N− N is the number of amino acids in the chain.

The R distribution, in contrast to the T distribution, represents a state with a uniformly distributed level of hydrophobicity (there is no hydrophobic core).

By comparing the values of D_KL_(O|T) and D_KL_(O|R), the ‘proximity’ of the two distributions being compared can be determined. The relation D_KL_(O|T) < D_KL_(O|R) indicates the presence of a hydrophobic core. To avoid using two values to describe the same object, the quantity ‘RD’ (Relative Distance) is introduced:

4 $$RD = \frac{{D_{KL} (O|T)}}{{D_{KL} (O|T) + D_{KL} (O|R)}}$$

An RD value < 0.5 indicates the presence of a hydrophobic core (Fig. 1B).

The deviations (the O distribution versus the T distribution) identified in protein distributions can be localised, where single and easily identifiable residues show a deficit or excess of hydrophobicity, as the case may be. If located in the common region of the protein molecule, residues with a hydrophobicity deficit in most cases are components of the active centre. Hydrophobicity deficits are often cavities ready to bind the ligand or the substrate (in the case of enzymes) [46]. A local hydrophobicity excess suggests the site of complex formation of a different protein [47]. Proteins with an O distribution similar to that of the T distribution were also identified. These are proteins with micelle-like structuring: down-hill, fast-folding, ultra-fast-folding and antifreeze type II [48].

The aqueous environment is not the only environment for protein activity.

Membrane proteins exhibit activity in a hydrophobic environment. Their stabilisation in this environment is ensured by the exposure of the hydrophobic residues to the outside (providing a preferable system with the hydrophobic membrane). If, in addition, the protein acts as an ion channel it has a concentration of polar residues in the central part (in particular). It is therefore an ‘inverted’ system in relation to proteins active in aqueous environments. Therefore, the idealised hydrophobicity distribution for the membrane environment is expressed by the complement of the 3D Gaussian function according to the equation below:

5 $${\text{M}}_{{\text{i}}} \, = \, \left[ {{\text{T}}_{{{\text{Max}}}} {-}{\text{T}}_{{\text{i}}} } \right]_{{\text{n}}}$$

where T_Max_ is the maximum value for the 3D Gaussian distribution and n is the normalisation of the resulting distribution.

As previous analyses showed, the proteins do not demonstrate an arrangement that follows the given distribution (Eq. 5). It appears that the proteins represent a structure that is a sort of consensus between the two forms, the Ti-compatible form and the Mi-compatible form. Therefore, the final distribution is determined by the following equation:

6 $${\text{M}}_{{\text{i}}} = \, \left[ {{\text{T}}_{{\text{i}}} + {\text{ K}}*\left( {{\text{T}}_{{{\text{Max}}}} - {\text{T}}_{{\text{i}}} } \right)_{{\text{n}}} } \right]_{{\text{n}}}$$

The K-factor indicates the degree of contribution of the ‘inverted’ distribution to the distribution expressed by the 3D Gaussian function (denoted here as T). This parameter expresses the strength with which a given environment modifies the system resulting from the polar water environment. The proper K value for particular set of T and O profiles is found as expressing the lowest D_KL_ (O|M) value (Fig. 1C). Finally the representation of M profile for T and O is shown in Fig. 1D.

More on membrane protein structure analysis is provided in [34, 49].

The graphical visualisation of the model in question (Fig. 1) illustrates the significance of the individual parameters and their interpretation.

The M distribution is therefore considered to be an expression of the effect of the environment on the protein folding process, in which the protein adapts to the conditions imposed by the environment.

If a juxtaposition of the distributions (Fig. 1) represented a hypothetical protein, this protein would be classified as deprived of a hydrophobic core.

It is also possible to perform an operation to eliminate the positions with the greatest differences between T_i_ and O_i_. A multiple step-wise elimination of these positions allows the identification of that part of the protein that exhibits RD < 0.5, thus identifying the part of the protein with a micelle-like organisation responsible for the solubility of the protein in question.

The summaries of successive editions of the CASP project show a split between ‘easy’ and ‘hard’ proteins. This distinction applies to all calculation techniques used by the participants. Traditionally, a high degree of prediction difficulty is associated with the presence of a beta-structure, which is more challenging (‘harder’) for obvious reasons (a long-range interaction). However, there are examples of proteins entirely representing the helical structure with a low prediction accuracy score. The search for an answer to the question posed earlier as to why very good force fields that provide models with a high degree of similarity to the target fail for other proteins is done based on the identification of the environmental differentiation of protein folding conditions.

### Programs used

The potential user has two possible ways to access the program:

The program allowing the calculation of RD as well as T and O distribution is accessible upon request on the CodeOcean platform:

https://codeocean.com/capsule/3084411/tree. Please contact the corresponding author to get access to your private program example.

The application—implemented in collaboration with the Sano Centre for Computational Medicine (https://sano.science) and running on resources contributed by ACC Cyfronet AGH.

(https://www.cyfronet.pl) in the framework of the PL-Grid Infrastructure (https://plgrid.pl)—provides a web wrapper for the abovementioned computational component and is freely available at https://hphob.sano.science.

The VMD program was used to present the 3D structures [50, 51].

## Results

### Selected models in the CASP13 project

The selection of examples for a detailed analysis was driven by the highest, medium and lowest model-to-target fit that ranked at the top of the list (per GDT_TS). The evaluation criterion used in the current work is the parameters of the FOD model (Table 1).

**Table 1:** Values of RD and K parameters for models that had the top position on the ranking list (the GDT_TS values)

TARGET			BEST MODEL			Correlation coefficient
ID	RD	K	K	RD	GDT_TS	
T0990-D3	0.528	0.5	0.6	0.575	48.71	− 0.275
T1024	0.648	0.8	0.4	0.540	63.30	− 0.466
T0953s-D3	0.286	0.1	0.3	0.468	43.01	− 0.785

The determination of RD and K values for the target – the T and O distributions obtained for the structures as given in PDB. The correlation coefficient expresses the relationship of the rating (per GDT_TS) of the model to the RD parameter value, which determines the degree of adaptation of the protein structure to environmental conditions

The interpretation of the respective sets as given in the columns is as follows:

- The TARGET column: the parameter values characterise the structure that is available in the PDB. According to the interpretation based on the FOD model, the RD value reveals the degree of organisation of the hydrophobicity distribution against the micelle-like distribution. The K parameter indicates the contribution of non-aqueous factors that affect the formation of the structure of the protein in question.
- The BEST MODEL column represents the status of the structure of the model by assessing the extent to which the T distribution is reproduced by the O distribution as represented by the model structure.
- The correlation coefficient: the relationship of the GDT_TS rating value to the status of all models expressed by the RD parameter values.

In the presented system, T0953s-D3 shows a structuring consistent with a micelle-like distribution (very low RD and K values). The structure of this protein is distinguished by the presence of a hydrophobic core and a polar surface. The contribution of non-aquatic factors is negligible. The values describing the status of the model ranked at the top of the GDT_TS classification also show a high adaptation to a micelle-like system, albeit significantly lower than the target status. This difference turns out to be significant, as the model is very poor in the assessment, despite its top position on the ranking list.

The model structure obtained for the target T1065s2-D1 turns out to be very close to that of the protein. Here, the best model scored very highly on the GDT_TS scale. The assessment based on the RD and K parameters also shows considerable similarity.

The target T0990-D3 shows structuring above the cut-off level (RD = 0.5) to a small extent (K = 0.5). The status of the proposed model appears to represent a distribution further away from the micelle-like system (higher RD and K values for the model against the target status). This difference results in a relatively low rating on the GDT_TS scale.

The target T1024 shows the highest variation against the top model in terms of RD and K. The top model was ranked with a relatively high GDT_TS value (63.3).

**Fig. 2:**
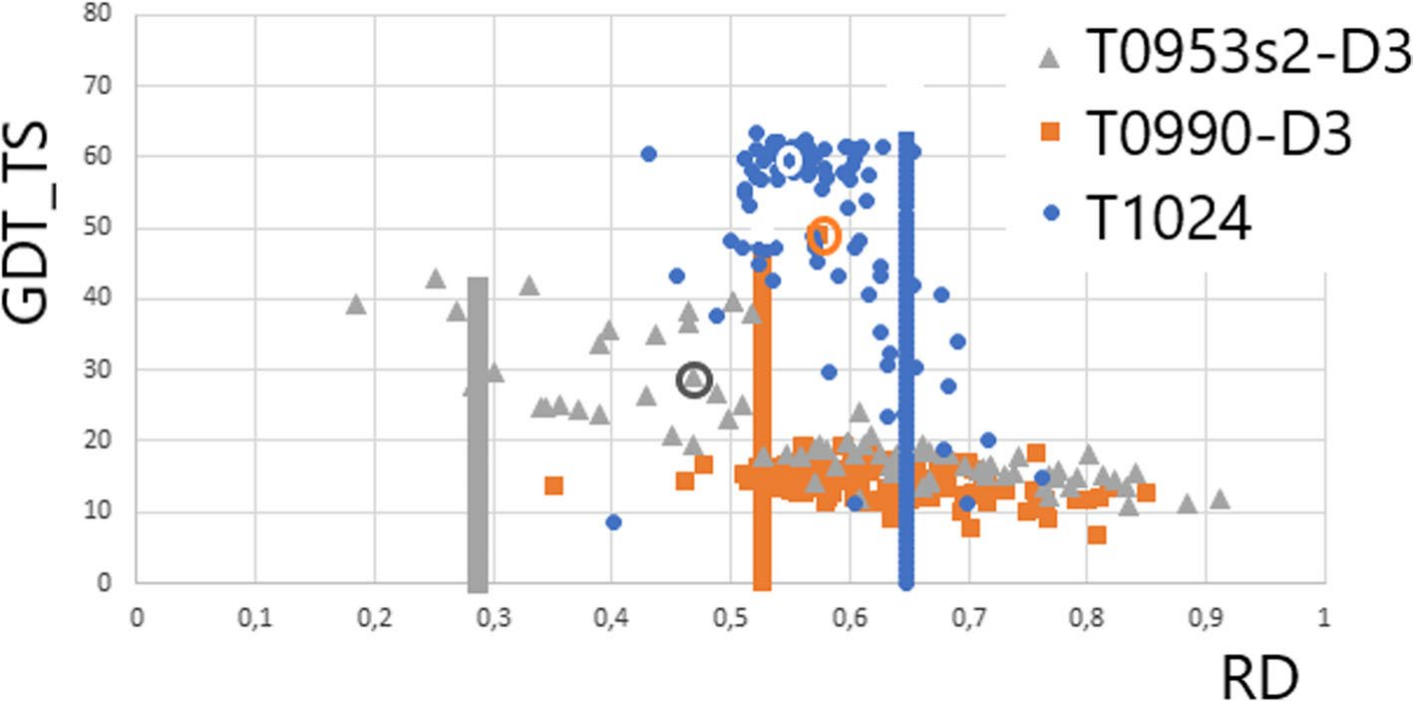
Dependence of the GDT_TS scale score on the status of the model protein structure expressed on the RD scale. The vertical lines are the RD values for the targets. The height of the vertical lines is the maximum score level on the GDT_TS scale. The encircled positions are the results obtained with AlphaFold

The meaning of the ‘Correlation Coefficient’ column (Table 1) is shown in Fig. 2 and Table 1 provides very similar RD and K values since the top models are presented. The spread of the analysis of the model statuses (on the RD scale) varies widely across all participants of the CASP. A dependence with a negative correlation coefficient value is revealed, expressing a decreasing score (GDT_TS) with an increasing RD that describes the status of the target. This means a lower rating (GDT_TS) for models that do not take into account deviations from the FOD-ordered distribution. The correlation coefficient value is variable and depends on the status of the target.

**Fig. 3:**
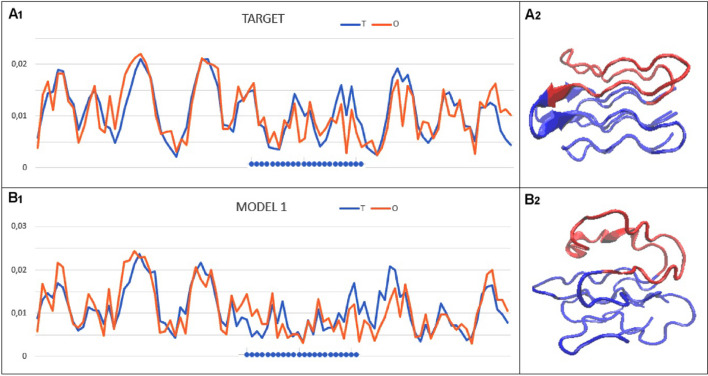
Example of incompatibility for T0953s2-D3. The blue dots on the x-axis identify the area that does not reproduce the arrangement present in the target protein. The 3D presentation with a highlighted section of the chain where a significant discrepancy between the top-ranked model against the target is present

The classical GDT_TS rating reduction relationship for RD values moving away from the target status is shown by the model obtained for the target T0953s2-D3 (Fig. 3), which is to the very low RD value that describes the status of the target. The relationship between the status expressed by RD and the GDT_TS assessment is expressed by the correlation coefficient equal to CC =  − 0.785 (Fig. 2). It means the larger the error in the RD status the lower the assessment of the model.

The positions circled in Fig. 2 are the results obtained with the AI method. It is apparent that the environment should also be considered in this method. This is particularly evident for the target T0953s2-D3.

As an example of the type of incompatibility between a model and a target in terms of the FOD model, the set of T and O profiles for T0953s2-D3 representing the lowest GDT_TS score is illustrated. Here, the degree of reproduction in the top-ranked model micelle-like system proved to be too poor. The central section of the chain (highlighted in red in the 3D image) in the proposed model contributes too little to the hydrophobic core.

To make the list of discussed examples complete, the structure of target T1065s2-D1 described by parameters: RD = 0.594 and K = 0.5 appeared to be very well predicted with GDT_TS score = 98.47. This best model for this target represents the structure of the status expressed by parameters RD = 0.578 and K = 0.5. This example proves applicability of RD and K parameters as possible criteria for structure comparison.

## Analysis of examples taken from CASP14 and CASP15

This analysis demonstrates the dependence of the result obtained (the top position on the ranking list) on the RD value of the target structure.

**Fig. 4:**
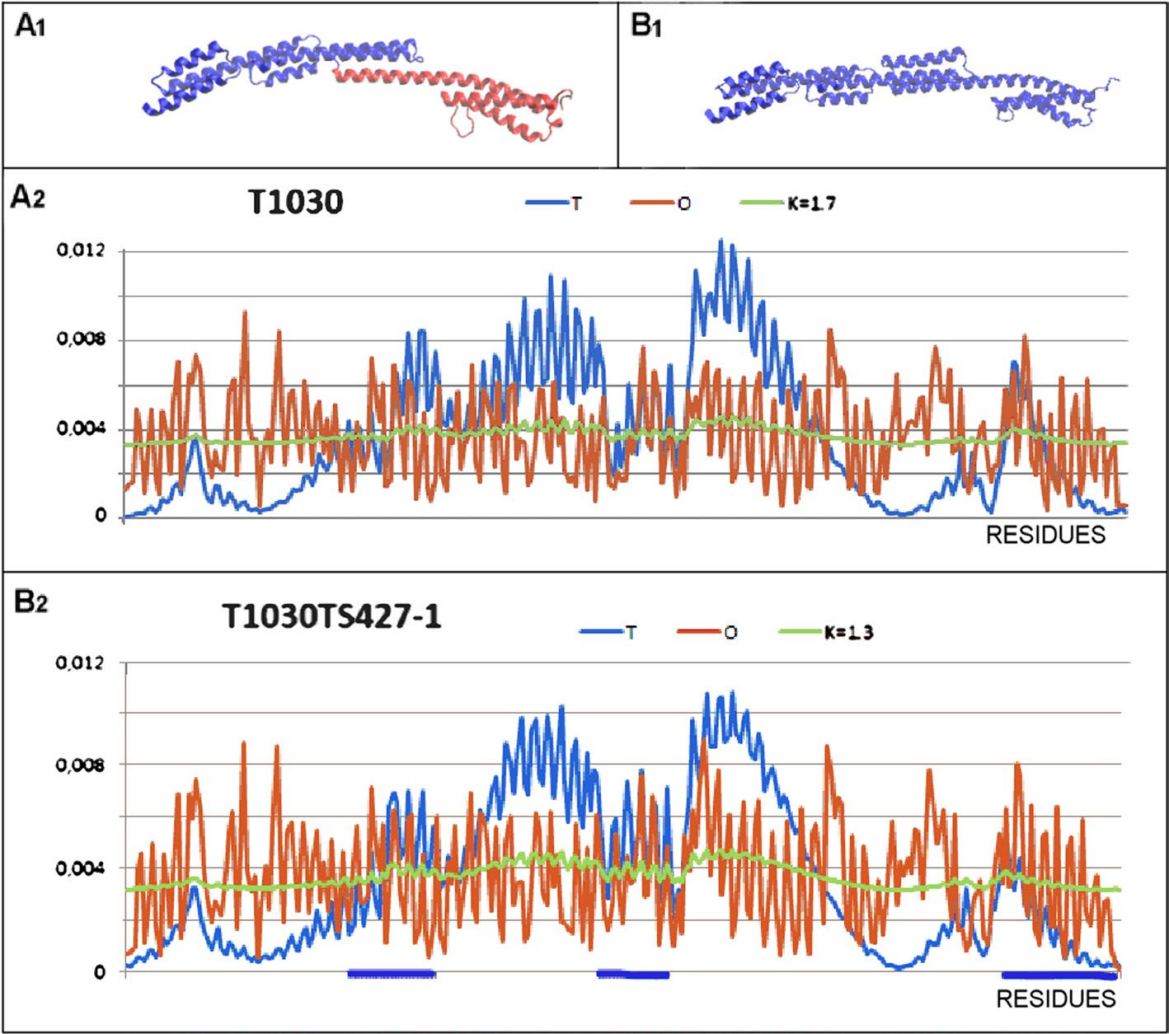
Characteristics of the N-terminal helical domain of biba, a group b *Streptococcus* immunogenic bacterial adhesin (PDB ID—6POO). **A** 3D structure left – the target T1030; right – the model T1030TS427-1 **B** set of T, O and M profiles for the corresponding K representing the target T1030 **C** set of T, O and M profiles for the corresponding K, representing the model T1030TS427-1

An extreme case of a globally distinct structure is a pathogenicity protein, putative from Streptococcus agalactiae serotype V from the *Streptococcus agalactiae* (PDB ID—6POO – target T1030 in CASP 14) [41] (Fig. 4).

The protein entirely represents an example of a helical protein with a structure far from globular. Therefore, a hydrophobic core is not expected to be present here. The structure of the protein (target) is described by the values: RD = 0.786 with K = 1.7 and, to determine the status of distinguished domains: (1–154) RD = 0.674 and K = 0.8, while for the domain (155–273), RD = 0.658 and K = 0.6.

High RD values indicate a structuring devoid of a hydrophobic core, while the K values suggest a significant contribution of factors that are not water, driving the structuring. A compilation of the T, O and M distributions for the protein in question shows a nearly linear distribution (similar to the R distribution). This type of distribution is interpreted to be a result of conditions where there is no impact of an aquatic environment. The closer to the horizontal line the M distribution is, the lower the influence the aqueous environment has on the structuring. A protein with this type of M distribution can be thought of as a protein folding effect in a specific ‘water void’ environment.

A summary of the results (Table 2) shows the lowest GDT-TS score for the complete chain. Considering the nearly complete helical structure of this protein, the result turns out to be very poor. The very high values of the K parameter with large differences for the model and the target reveal the need to diversify the environment (its influence on the target is much higher than that obtained for the model).

**Table 2:** Set of parameters based on the FOD model for the target T1030 and the model T1030TS427-1

	GDT_TS	RD-target/RD-model	K-target/K-model
D1	78.73	0.674/0.671	0.8/0.9
D2	89.5	0.658/0.640	0.6/0.6
CHAIN	63.0	0.786/0.757	1.7/1.3

An example that also requires a detailed discussion is the unknown function target category protein, T1029 (PDB ID—6UF2 [42]).

Complete with respective 3D presentations. Highlighted in red – fragments showing a hydrophobicity deficit; blue – hydrophobicity excess, as shown in the diagrams.

**Fig. 5:**
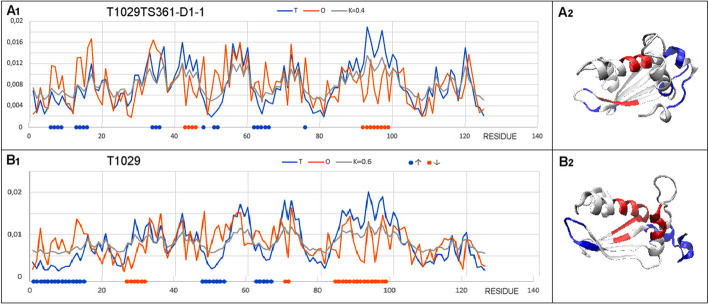
Set of T, O and M profiles for the corresponding K, representing **A** Model T1029TS361-D1-1 **B** Target T1029

The selected example represents the model with the lowest GDT_TS score in the set under discussion (Fig. 5). The parameters based on FOD-M for the target molecule are RD = 0.622 and K = 0.6 and GDT-TS = 45.8, with RD = 0.622 and K = 0.4 for the model. The assessment of the accuracy of the model structuring according to the criteria based on the FOD model is higher against a very low score on the GDT_TS scale. Rather, the identical RD values and the low variance of the K values suggest the accuracy of the model structure. When analysing the T, O and M profiles, a peculiar notation of property variation is seen, suggesting possible biological activity. It is indeed possible to identify a chain fragment whose status, revealing a local hydrophobicity deficit, suggests the presence of a cavity (the red fragments in Fig. 5) ready to interact with the ligand. It is feasible to speculate on a possible interaction with another protein via an N-terminal chain fragment showing excess hydrophobicity. High RD and K values may suggest the need for other factors besides water to contribute. The location of sections of the O distribution significantly diverging from the T distribution suggests a similar design of the potential ligand binding cavity.

### Comparative analysis of results obtained by the same force field – Baker-Rosettaserver (participant No. 368) (CASP13)

The model proposed in a group of programs whose history began with the ROSETTA program [52] is represented in a subsequent version modified in the form available with the Baker-Rosettaserver. The force field used in this program package is known very well. Alongside numerous successes, the group also delivered poorly rated results. It is therefore possible to pose the question as to why a very good force field fails in some cases. The search for an answer to this question was based on the analysis of a set of results in the form of models provided under ID 368 Baker-Rosettaserver in the CASP13 project [43].

**Fig. 6:**
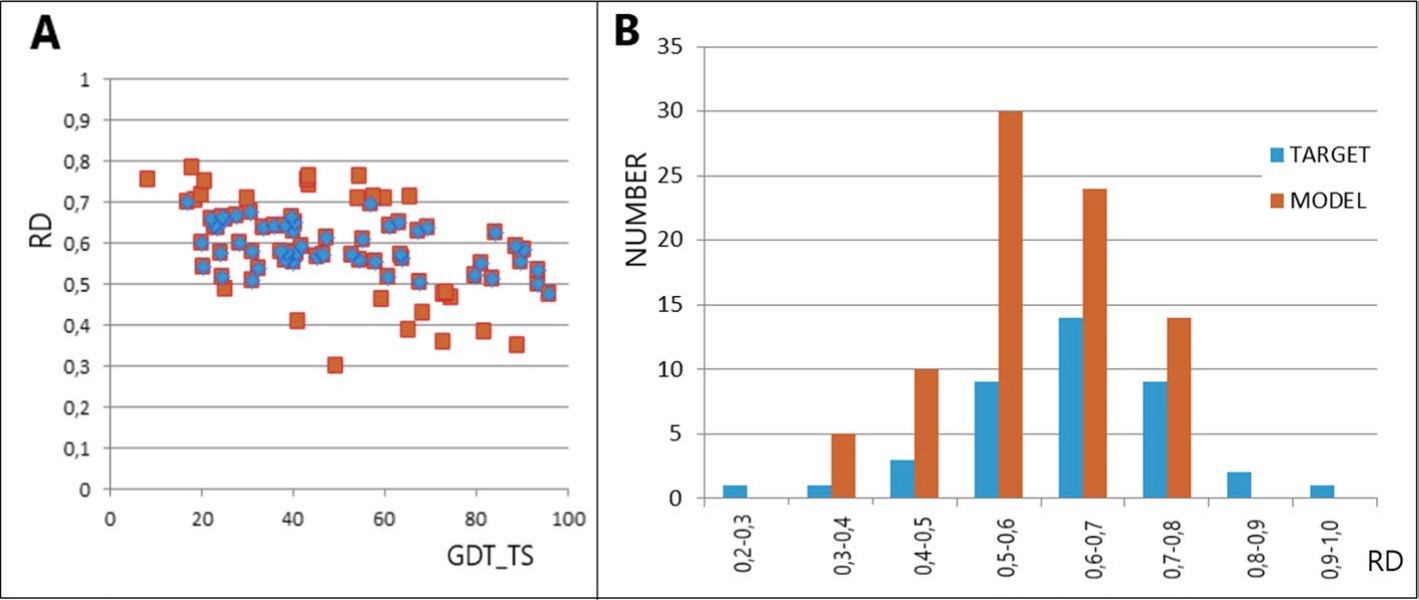
Analysis of the models obtained with the Baker-Rosettaserver **A** relationship of the RD value to the GDT_TS score. The blue dots represent the range of RD of highest representation as shown in B. **B** number of available targets and delivered models for the RD value ranges

The results of the evaluation of the models obtained with this server are presented in Fig. 6.

Analysis of the set of results (Fig. 6A) shows a correlation coefficient of -0.562. This is a result of very low scores for target protein status models with high RD values and two relatively good scores for low RD values. The summary in Fig. 6B reveals the status of the target proteins and the status of the models provided. The most numerous group of models represents the RD range of 0.5 < RD < 0.7, while the 0.6–0.7 range is the most numerous in the target group. This abundant presence is representative of the entire pool of proteins (an opinion expressed on the basis of the analysis of numerous proteins available in the publications of the I. Roterman team – the results have not been published). It can be speculated that the parameterisation used in the programs in the ROSETTA group (in particular the one used in the Baker-Rosettaserver – participant No. 368) was determined based on the analysis of multiple proteins, which justifies representing only this range of RD values.

The summary in Fig. 6B reveals the absence of models with extreme statuses – low and high RD values. This observation reveals the need to vary the force field used to predict protein structures.

Of particular note are two structures (PDB ID–6CL6 [53] and PDB ID 6F45 [37]) whose native form shows extreme values of K = 4.0 for 6CL6 and K = 0.1 for 6F45. These examples represent cases that are in the target form but absent in the models provided (Fig. 6B) (with an RD-based classification).

**Fig. 7:**
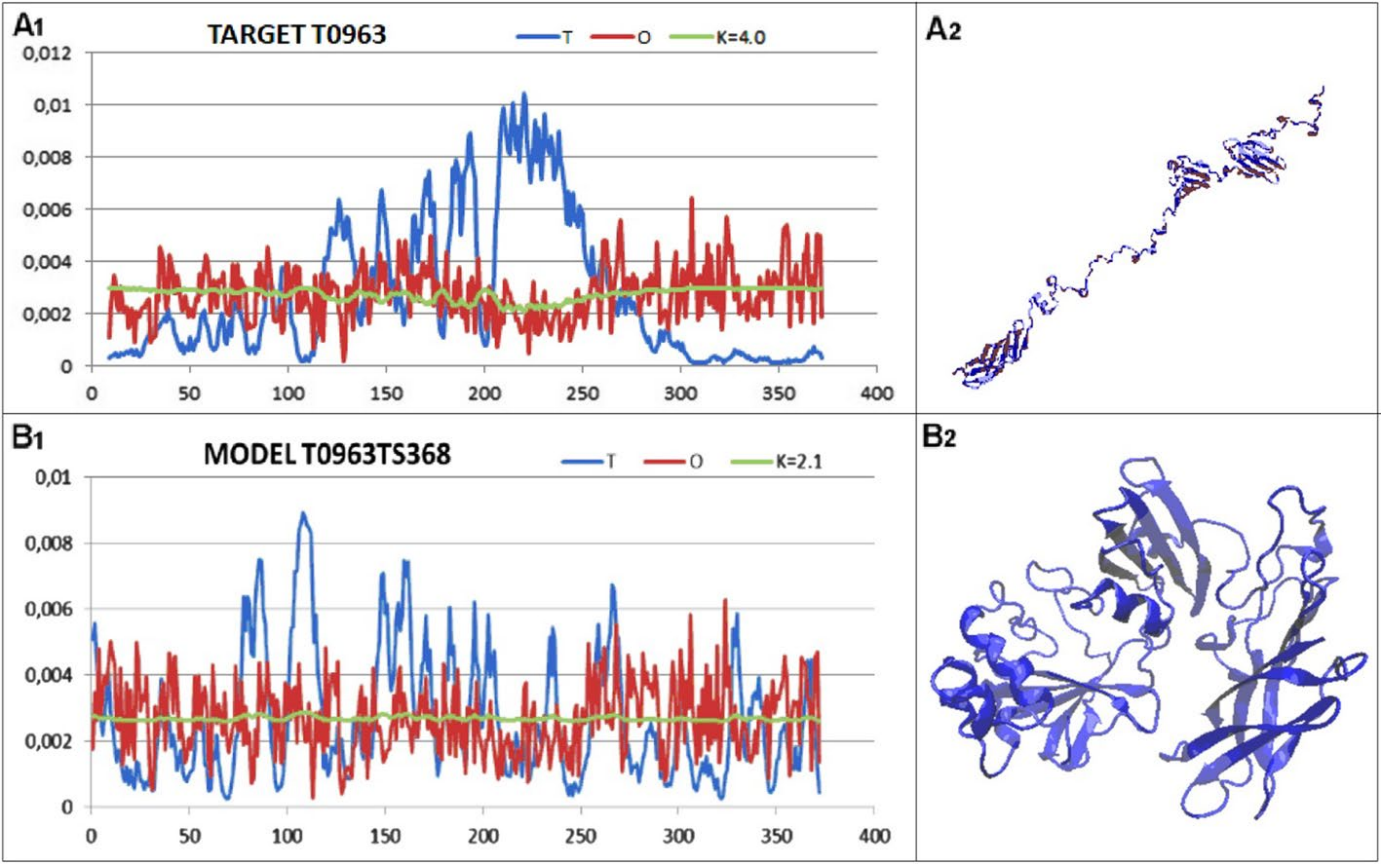
Summary of the T, O and M profiles for the corresponding K values. **A** Target T0963 together with the 3D structure of the native form of the protein in question (PDB ID 6CL6) **B** – Model T0963TS368 together with the 3D structure proposed using the Baker-Rosettaserver (participant No. 368) [43]

Analysis of the T, O and M profiles reveal the cause of the different structures of the target T0963, and the model provided by the server in question (Fig. 7). Notable is the fundamentally different RD value, with the target protein structure showing a significantly higher value. In contrast, a significantly higher value for the K parameter reveals the role of the environment. The target structure requires a suitable environment to stabilise this non-globular structure. It is clear that this structure would not be formed in an water environment. This is particularly evident in the N-terminal sections and particularly in the C-terminal sections, which show significantly higher hydrophobicity than the superficial location of these sections would suggest. This represents a preparation for interactions with other chains, which is in fact what happens when studying the biological activity of this protein [53]. This example clearly reveals the need of considering the presence of a certain external ‘rack’ in the form of non-aqueous factors stabilising this structural form that is far from globular.

**Fig. 8:**
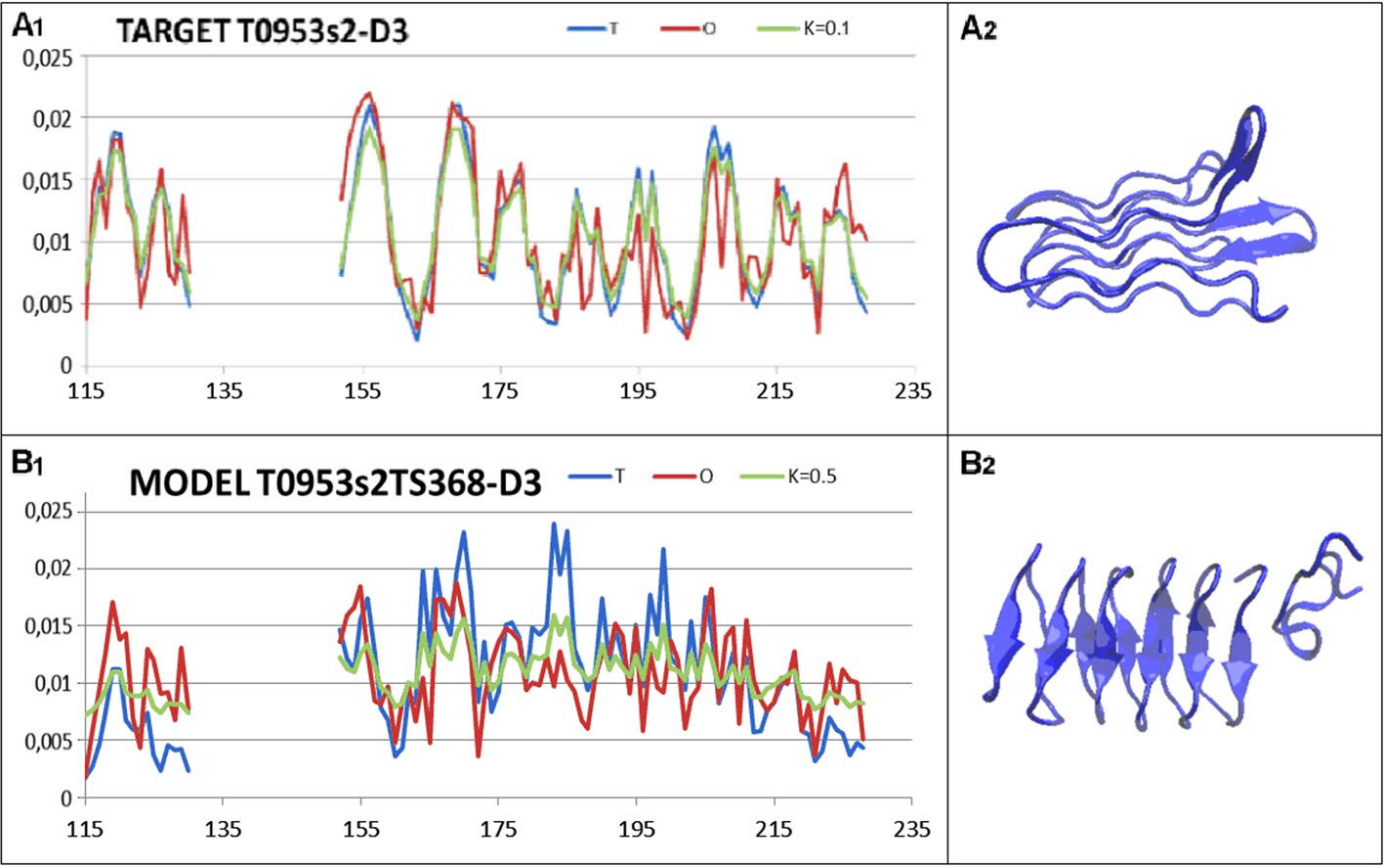
LOW A set of the T, O and M profiles for the respective K values, together with a 3D presentation for: **A** target T0953s2-D3 **B** model T0953s2TS368-D3

A second example of the target whose status was not reproduced in the model is the target T0953s2-D2, due to its very low RD (target: 0.286) at RD (model: 0.522) (Fig. 8).

In the set of targets in the CASP13 edition, the example representing a micelle-like structure is the D3 domain (115–130, 152–228) (PDB ID 6F45). The RD value for the target is 0.286 (extremely low) with K = 0.1, while the structure predicted by T0953s2TS368-D3 shows a structuring with RD = 0.599 and K = 0.5. This is an example of a structure determination by a force field, whose parameterisation is focused on the range of 0.5 < RD < 0.7, as shown earlier. Here, the structure of the model could probably be reproduced by running simulations of the protein folding process in an aqueous environment that directs the process towards a micelle-like form [35].

## Discussion

The vast majority of programs in the ab initio category as well as the recently introduced AI-based method are driven by parameterisation resulting from the analysis of protein structures available in the PDB. The proteome of a particular organism is made up of proteins performing all the biological activities required to sustain life. Given the complexity of the system that is the world of living organisms, it is not surprising and indeed seems necessary to have a high degree of diversity in the tools that are proteins. Structural differentiation stems from the differences in amino acid sequences. In addition, the environment in which a protein performs its function is also a source of high diversity in the protein world. The environmental diversity has a very wide range, which provides a highly differentiating factor for the structures obtained. Therefore, a force field that does not consider external conditions or refers to ‘averaged’ characteristics remains deficient in relation to the great variety of tools and machines that are the proteins in every living organism.

**Fig. 9:**
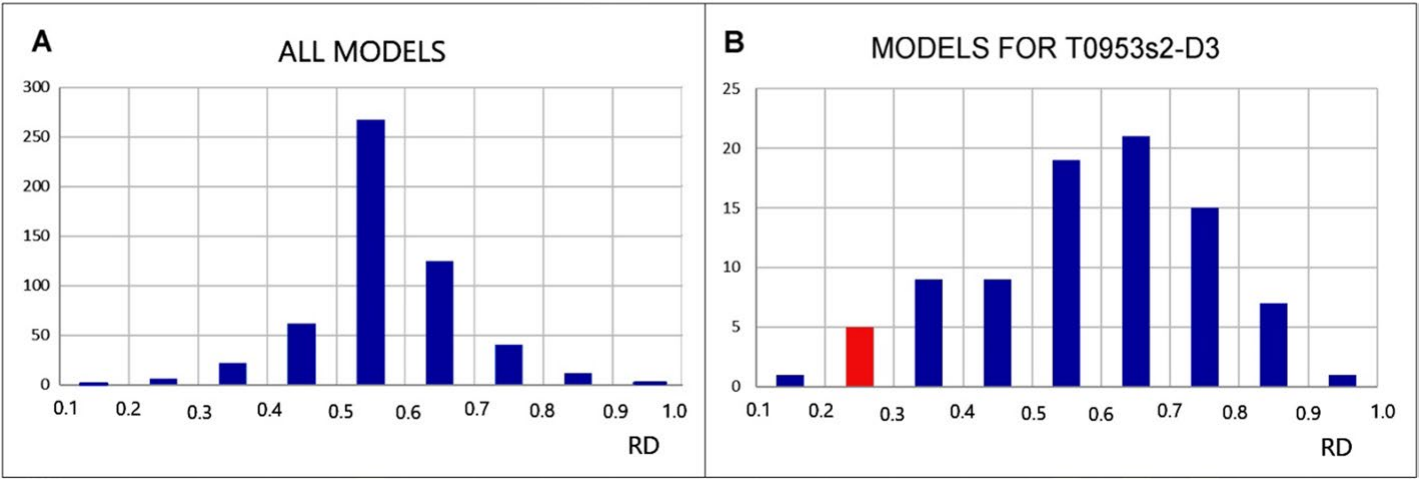
Summary of the results based on the examples discussed. The vertical axis expresses the number of models provided for a given range of RD values. **A** all examples discussed in this work. **B** target T0953s2-D3 representing the lowest RD = 0.286 for the target status – the range distinguished by the red bar

The characterisation of the target T0953s2-D3 (CASP13), the structure of which no method could handle, shows a very low value of the K parameter = 0.1 in the native structure. This means that the force fields applied (which presumably are averaged) cannot reproduce the structure according to the micellization mechanism. As shown in Fig. 6 and Fig. 9, the range of characteristics best reproduced in the programs is between RD 0.5 and 0.7. According to the assessment to date, this is the range most highly represented by proteins of the proteome. Within this range are single-chain enzymes less than 200 aa in length with a clearly localised incompatibility with the micelle-like system (an active centre). Enzymes with an extended structure (300–500 aa chains) and specific incompatibility with the micelle-like system are also in this range. The incompatibility consists of a scattering of small differences along the chain, without being able to distinguish the specific location of an explicit micelle-like incompatibility. This numerical predominance of proteins with the status expressed as 0.5 < RD < 0.7 indicates the reasons why the largest number of highly rated models precisely involve structures that, under conditions of activity, represent such a structure.

The need to consider environmental specificity for the folding protein is revealed by all amyloid proteins, whose change in structure is not caused by mutation but, it seems, solely by environmental effects.

The proposal to include a structure assessment in the RD and K category was already raised [54]. Structures rated highly by geometrical criteria (GDT_TS) do not perform well in terms of specificity derived from the hydrophobicity system (RD and K), which is important for the biological functions performed by proteins.

A summary (Fig. 9) reveals the dominance of the parameterisation of the tools used over the range of variation in environmental conditions expressed as 0.5 < RD < 0.7. According to analyses carried out by the authors of this work, this is the most common range of variation for this parameter. If the parameterisation for the tools used had been based on domain structures, the range would have been expressed with much lower RD values, as the domains show a large majority of structuring based on the presence of a hydrophobic core (low RD and low K values).

The summary of results (Fig. 9) reveals a dominant parameterisation for structures defined by the FOD-M model convention as representing a status with 0.5 < RD < 0.7. This coverage is mostly present in large-scale analysis of proteins as available in PDB. The standardisation of force fields and other criteria for protein structure prediction seems to be common for all procedures independently of the method applied. The data base in the form of domains as the test set for parameterisation shall deliver the results from the range 0.0 < RD < 0.4 since this range has been identified for large scale analysis of domains as they are available in PDB.

In conclusion: the protein structures do not follow the averaged model. The differentiation of force fields expressing the external conditions shall be present in models oriented on protein structure prediction.

The proteins presented in this analysis were selected subjectively, focusing mainly on examples that show significant differentiation in performance between groups and examples that score low, despite the use of models that perform very well for other targets.

The analysis of two examples with extreme RD values (0.286 and 0.918), whose structures were predicted to have RD values of 0.599 and 0.783 (with GDT_TS scores of 43.01 and 24.66, respectively), demonstrate the validity of the thesis assumed here about the necessity of the environmental factor.

## Conclusion

As long as protein structure prediction-oriented programs do not take into account the presence of environmental conditions, the prediction is only reliable for a limited group of proteins (here defined by parameters based on the FOD model at 0.5 < RD < 0.7). The use of an averaged parameterisation determined from the analysis of known structures cannot lead to the prediction of protein structures, which are themselves differentiated as products of the contribution of the environment directing the protein folding process.

This work proposes a model that takes into account the environmental specificities to be considered in the construction of the external force field.

An external field for successive values of K (from K = 0 to K = 1.0 and even K > 1.0) applied to an energy optimisation procedure extended by optimisation due to the presence of an environment that directs the protein folding process appears to be necessary. An unambiguous example is amyloid proteins, which, with an unchanged sequence, acquire very different structural forms depending on the environment (shaking as the experimental procedure for amyloid production). These conclusions are also confirmed by the WeFold project, where combining the tools of the leading groups did not significantly change the validity of the results obtained [23]. The example shown in [35] also supports this conclusion.

## Data Availability

All data can be available on request addressed to the corresponding author. The program allowing calculation of RD is accessible on GitHub platform: https://github.com/KatarzynaStapor/FODmodel and on the platform https://hphob.sano.science.

## Abbreviations

PDB: Protein data bank
CASP#: Critical assessment of structure prediction with appropriate number of project edition
RD: Relative distance
GDT_TS: The name of scale applied by CASP model to express the degree of model-target similarity–Global Distance Test – Template Score
